# Writing for a Doctor of Philosophy (PhD): A guide to developing a strong and coherent thesis

**DOI:** 10.4102/phcfm.v17i2.5164

**Published:** 2025-11-29

**Authors:** Mergan Naidoo, Kimera T. Suthiram, Luckson Dullie

**Affiliations:** 1 Department of Family Medicine, College of Health Sciences, University of KwaZulu-Natal, Durban, South Africa; 2 Department of Family Medicine, Faculty of Medicine, Kamuzu University of Health Sciences, Blantyre, Malawi

**Keywords:** PhD thesis writing, academic argument, doctoral education, synthesis chapter, theoretical framework, conceptual framework, thesis structure, scholarly identity, higher education

## Abstract

Academic writing is a central yet often under-estimated component of doctoral education. More than a mechanism for transcribing research findings, writing is a generative and iterative process through which doctoral candidates cultivate a scholarly voice, construct persuasive arguments and make an original contribution to knowledge. This article provides a comprehensive guide to writing a coherent and compelling Doctor of Philosophy (PhD) thesis, offering both conceptual clarity and practical strategies. The article begins by exploring how doctoral candidates can develop authoritative academic writing, with emphasis on writing style; consistency, coherence and cohesion; the iterative writing process; and how to develop argumentation. The article outlines the typical structural formats of the PhD thesis, with a focus on both the traditional monograph and the increasingly common thesis by publication. Particular attention is given to the integration of conceptual or theoretical frameworks, research paradigms and study design, and analytical frameworks – ensuring that research design is underpinned by methodological rigour and philosophical consistency. A dedicated section offers guidance on writing the final synthesis chapter, detailing approaches for critically engaging with findings, connecting them to theoretical perspectives and articulating their contribution to the field. By demystifying the thesis writing process and offering actionable insights, this article aims to empower doctoral candidates to write with clarity, confidence and scholarly rigour – ultimately producing a thesis that reflects academic maturity and meaningfully advances their discipline.

## Introduction

Academic writing enables doctoral candidates to articulate original ideas and theories in an in-depth thesis or scholarly articles. At the doctoral level, the thesis is expected to demonstrate: (1) sufficient scope, typically equivalent to a body of work that could generate several publications without salami slicing; (2) originality, contributing new knowledge at the cutting edge of the discipline with international value and (3) academic rigour, showing scientific validity and methodological robustness. These elements are assessed both at the stage of protocol registration and in the final thesis. While research is often seen as the generation of new knowledge, writing is frequently perceived as a mere ‘write-up’ of that process.^1^ This reductive view undermines the significance of academic writing and diminishes its vital role in doctoral education. Scholarly writing is a fundamental skill that allows doctoral candidates to think about their research and communicate their research clearly and persuasively to a broad audience, contributing to advancing theory and knowledge within their field.

Doctoral writing is, therefore, central to becoming doctoral.^2^ A growing body of research highlights the developmental impact of doctoral education on students’ academic identities. Some studies emphasised that the development of a doctoral candidate is a socially embedded process.^3,4^ Other studies underline how academic writing plays a crucial role in enabling students to construct their scholarly voice and identity, thereby establishing themselves within academic communities.^5,6^ Academic writing encapsulates more than learning how to master language; it is about learning to participate in a community of practice, negotiating one’s identity and navigating institutional and cultural norms.

Despite its importance, academic writing can be daunting for many doctoral candidates, particularly those in the early stages of identifying as independent researchers and scientific writers.^2^ Apart from the intellectual and conceptual labour that is central to doctoral work, students often struggle with the lack of clear guidelines on how to organise and structure their thesis, as well as with determining what content is most relevant and essential. Common challenges include losing momentum, fatigue, writer’s block, fear of failure, anxiety over feedback and dissatisfaction with writing quality. To address these issues, this article offers practical guidance to help doctoral candidates build the necessary skills to become confident, authoritative academic writers. It highlights strategies for constructing logical and coherent arguments, integrating philosophical and theoretical perspectives and effectively structuring the thesis, particularly the synthesis chapter.

## Becoming an authoritative writer and building your argument as a Doctor of Philosophy (PhD) scholar

### Academic language and style

Mastering the language and style of academic writing is essential for fostering credibility and establishing scholarly arguments. Scholarly discourse requires a formal tone that reflects the significance of a contribution to its respective field. Skilled academic writers avoid colloquial expressions, contractions, excessive use of personal pronouns and informal language.

While grammar, structure and sentence construction are fundamental components of academic writing, effective scholarly communication extends beyond these mechanics. Academic writing does not need to rely on long, complex sentences filled with multiple subclauses or unnecessarily sophisticated language. While technical terms are sometimes essential, sentences should remain clear, concise and easy to understand. Effective academic writing uses precise language and focuses on a clearly defined area of inquiry.

### Consistency, coherence and cohesion

Ensuring alignment between research questions, objectives, hypotheses and methods significantly enhances the consistency, coherence and cohesion of a thesis.^7^ Consistency ensures methodological uniformity by making use of the same terminology, formatting and style throughout the thesis to prevent contradictions, while coherence refers to the logical structuring of arguments across the thesis, with each section or chapter connecting to the next. Cohesion involves linking ideas within and between paragraphs to maintain clarity and flow. Well-organised paragraphs begin with topic sentences that define the scope of that paragraph and the logical link to the previous paragraph, which are supported by facts and explanations, allowing readers to follow the logical progression from claim to evidence.^8^ Writers must also maintain objectivity, ensuring statements are grounded in empirical evidence rather than personal beliefs. Excessive self-referencing and over-citation should be avoided, as these practices can undermine credibility and disrupt the flow of the argument.

Transitional devices and connectors are essential for maintaining argumentative flow and ensuring continuity of thought.^8^ Forecasting and signposting are strategies that further improve coherence and readability. Forecasting outlines the main points to be discussed and helps readers anticipate the direction of the argument.^8^ It transforms a complex set of ideas into a logical progression of thoughts, demonstrating the student’s level of understanding of the research and providing the reader or examiner with a structured, purposeful inquiry. Effective and consistent forecasting allows the examiner to anticipate the main findings and conclusions. Signposting uses specific words or phrases to indicate transitions or relationships between ideas, guides the reader through the structure and reinforces the thesis statement.

### The iterative writing process and feedback

Writing is an iterative process that evolves through continuous drafting, feedback and revision. Fluency and quality emerge through consistent practice and the refinement of ideas.^8^ Through these cycles of writing, doctoral candidates develop a distinct academic voice and move closer to producing work that meets the highest standards of academic excellence.

The foundation of any writing is planning, which involves defining the argument and creating a detailed outline for each subsection or chapter of the thesis. The writer should outline key points in a logical sequence that can later be expanded into paragraphs. Such planning can be carried out as rough work in a notebook or journal and is usually a personal hidden part of the process.

Drafting is essential in expanding the plan into actual text, articulating your ideas and presenting your findings. At this stage of the writing process, elements such as grammar, writing style and sentence construction are not prioritised. The logical structure and clear content should be present, with attention being paid to the target audience.

Lastly, editing involves revising the text for issues such as spelling and language style, grammar and consistent formatting of headings and titles. The writing is proofread for errors and inconsistencies in references and formatting. Your supervisor will be instrumental in the drafting and editing stages to provide constructive feedback and guide the development of the writing in an iterative process that can take several weeks.

### Argumentation based on the literature

Use of the literature in scholarly writing should progress from listing, to synthesising and analysing, to finally authorising the argument that you want to make. Novice researchers often summarise and list the literature they have read article by article in a sequence of paragraphs. As they progress, they learn to organise or synthesise the literature by categories. For example, from global to local studies or from older or newer articles. Following this, the researcher may start to apply their skills in critical appraisal to analyse the strengths and weaknesses or contradictions of the evidence contained in these various articles. They may start to place more emphasis on the stronger evidence. All such writing tends to be descriptive of the literature and may lack a clear purpose in the overall logic of the thesis.^9^

Finally, the doctoral researcher learns how to integrate all the evidence into an argument that makes sense of the literature and has a purpose in the structure and flow of the thesis. The literature is gathered, appraised and used to support the argument appropriate to that part of the thesis. For example, the argument for the social or scientific value of the work, or for the conceptual framework, or the implications of the findings.^10^ Literature is not reviewed without a purpose, but is harnessed in support of the thesis.

A well-defined thesis statement may be helpful as a navigational tool, guiding the reader through the evidence underpinning the core argument.^11^ Such a statement at the end of a subsection or chapter can summarise the main points and keep the reader on track with the overall logic of the thesis.

A scholarly argument comprises three primary components: the claim, the evidence and the explanation. The writer may make a claim and reference the evidence that supports this claim. All such claims or statements should be supported by evidence that is cited in the text, unless the statement is clearly your own opinion. Evidence should be from primary sources that directly support the claim. Further explanation or elaboration may qualify the claim, recognising the strengths and weaknesses of the evidence, or acknowledging reservations because of contradictory evidence and exceptions.^12^ Engaging with counter-arguments also reflects intellectual rigour and strengthens the author’s stance within scholarly debates.^11^

Ultimately, the writer is taking a position in the academic discourse through their analysis of the literature. In critically engaging with the literature, they develop their own scholarly voice within the broader academic discourse.^13^ You place yourself in your chosen research field.

### Avoid plagiarism and use artificial intelligence appropriately

Doctoral candidates often emulate academic styles to build confidence. However, quality scholarly writing emerges when students present original, evidence-based arguments that reflect independent and critical thought. Doctoral writers must avoid plagiarism by appropriately paraphrasing and citing sources. Increasingly, candidates may also encounter AI (artificial intelligence) tools in the writing process; while these can support tasks such as grammar checking, their use must be transparent and should never replace original scholarly reasoning or writing. Developing this capacity for constructing logical, coherent arguments is essential for building academic identity and confidence, ultimately culminating in a thesis that upholds the standards of academic excellence.

## Structuring your PhD thesis

Having established the importance of aligning conceptual, theoretical, design or analytical frameworks with the study’s philosophical underpinnings, it is equally critical to consider how this coherence is carried through in the structural organisation of the PhD thesis itself. Rather than treating the literature review as a stand-alone chapter, candidates should integrate the literature purposefully throughout their thesis. For example, literature supports the case for social and scientific value, informs the development of frameworks, makes sense of findings and strengthens implications. This approach prevents the literature from becoming a descriptive summary and instead makes it an active part of the thesis argument. In Southern African universities, two main formats are accepted: the traditional monograph and the thesis by publication (also known as a manuscript-based thesis). Institutional preferences vary, but the latter is increasingly emphasised to encourage research dissemination during candidature. Table 1 outlines the various formats.^14,15,16,17,18^

**TABLE 1:** Structure of a PhD thesis.

Core question	Essential component	Traditional thesis (chapter location)	Thesis by publication (chapter location)
Why is this research important?	Social value: relevance and importance of the topic (with literature)	Chapter 1	Introductory chapter
How do we make sense of this topic?	Conceptual or theoretical framework (with literature)	Chapter 2	Introductory Chapter 1
What is the knowledge gap?	Scientific value: what we know and what we do not know (with literature)	Chapter 2	Introductory Chapter 1
What will be addressed?	Aims and objectives	Chapter 2	Introductory Chapter 1
How will this be addressed?	Study design and methods, analytical framework (with literature)	Chapter 3	Overall design in the introductory Chapter 1 and methods in individual articles
What are the findings?	Results	Chapter 4	Article manuscripts in Chapter 2
What do these results mean?	Interpretation, synthesis and relation to literature	Chapter 5	Article manuscripts in Chapter 2
What new knowledge emerges?	Conclusions (linked to aims/frameworks)	Chapter 6	Synthesis/Conclusions Chapter 3
What should be done with this knowledge?	Recommendations or implications (with literature)	Chapter 6	Synthesis/Conclusions Chapter 3
How will it have an impact?	Dissemination, future research/impact	Chapter 6	Synthesis/Conclusions Chapter 3

PhD, Doctor of Philosophy.

An increasing number of South African institutions advocate for the thesis-by-publication format, as it promotes research productivity, fosters early scholarly engagement and facilitates the timely dissemination of findings into the public domain. This format also enables candidates to progress in manageable, article-sized units. For many clinicians, this approach is particularly well-suited to balancing the demands of clinical work with doctoral study.^19^

Word count expectations vary across disciplines, institutions and formats. While some guidelines suggest a range of 15 000 to 30 000 words, this is more typical of highly focused, article-based theses in certain scientific disciplines. In fields such as health professions education or social sciences, traditional monographs often extend to 50 000–80 000 words, reflecting the more discursive nature of these disciplines and their broader engagement with conceptual and methodological debates. By contrast, theses in the health sciences are often more concise and tightly focused on methodological rigour and empirical findings, typically requiring fewer words to meet doctoral expectations. Table 2 provides approximate benchmarks for the thesis-by-publication format. It is important to observe that this format is typically much shorter than the traditional monograph, reflecting its focus on article-length units. This brevity makes the approach particularly appealing in Family Medicine and health sciences, where many candidates are clinicians who benefit from progressing in smaller, publishable segments.^14,15,16,17^ Most universities require standard front matter and supplementary material, as shown in Table 3.

**TABLE 2:** Typical word counts required for the thesis by publication.

Section	Typical word count (range)
Introductory chapter	~5500–8000 words
Manuscripts (combined)	10 000–25 000 words (depending on journal style)
Synthesis/Conclusion chapter	~2000–3000 words
Total (approx.)	~17 500–36 000 words

approx., approximately.

**TABLE 3:** Preliminary and supporting sections of a PhD thesis.

Section	Content
Title page	Thesis title, degree, candidate name, supervisors and format declaration (e.g. ‘Thesis by publication’).
Declaration	Confirms originality of the work.
Acknowledgements and Abstract	Summarise contributions and findings.
Lists	Abbreviations, tables and figures for navigation.
Ethics approval	Proof of ethics clearance (where applicable).
Plagiarism check	Verification of originality (Turnitin or equivalent).
Manuscript evidence	Proof of manuscript submission, acceptance or publication (for thesis by publication).
Appendices	Supporting material (e.g. data collection tools, supplementary analyses, letters).

PhD, Doctor of Philosophy.

Some universities also require ethics clearance documentation, plagiarism checks and proof of manuscript submission or acceptance. Always consult your university’s postgraduate regulations and supervisor for specific formatting requirements and policies. Candidates are encouraged to begin publishing early to ensure that manuscripts are ready or under review by the time of submission. The number of accepted or published articles required at submission varies by institution, and candidates should consult their university’s regulations for specific requirements. In multiarticle formats, particular care should be taken to ensure coherence across chapters and bridging texts, so that the thesis reads as a unified whole. Choose a format that aligns with your goals, institutional expectations and the nature of your research. With the structural components in place, the synthesis chapter becomes a critical space for integrating the various elements of the thesis – drawing together findings, theory and literature to demonstrate the study’s overall contribution to knowledge.

## Incorporating conceptual or theoretical frameworks

While the section above has focused on writing and the role that it plays in doctoral work, this section homes in on how the doctoral writer can incorporate conceptual, theoretical or analytical frameworks.

### Conceptual framework

**FIGURE 1:**
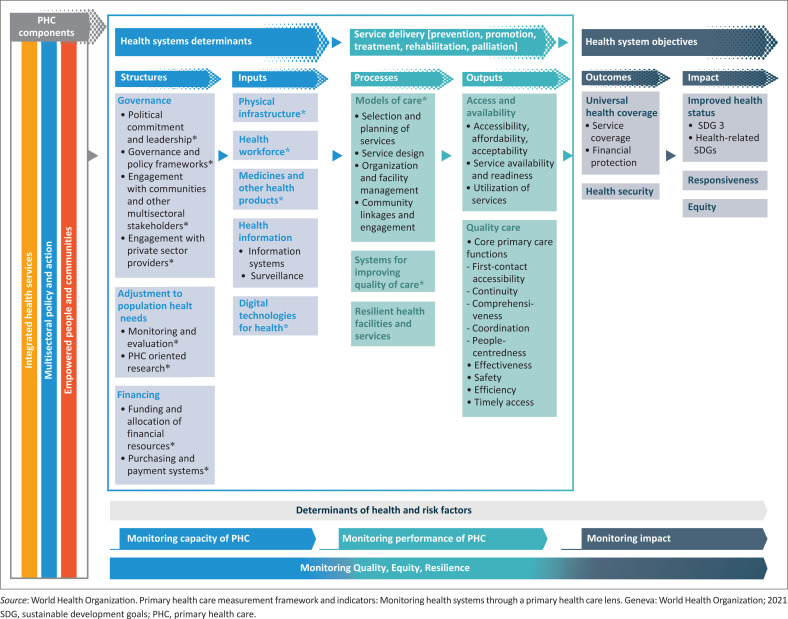
The World Health Organization’s conceptual framework of primary health care performance.

The conceptual framework outlines the key concepts and their interrelationships – often visualised through a model – and is derived from the literature, prior research and the researcher’s understanding of the phenomenon. Both conceptual and theoretical frameworks can provide a lens through which a study is interpreted. In many health science theses, the conceptual framework serves this role by mapping the relationships between key concepts and shaping the research focus. The conceptual framework emerges from critically engaging with literature and previous studies to map out how key ideas relate to each other in the context of the research problem. This framework is unique to each study and helps delineate the scope and focus of inquiry.^20^ Trafford and Leshem describe this as moving beyond a mechanical literature review to a ‘visual architecture’ that reveals the research’s intellectual map.^21^ An example of a conceptual framework is presented in Figure 1.^22^

The theoretical foundation rests on the primary health care (PHC) theory of change, which postulates that strengthening PHC – through integrated health services, multisectoral action and community empowerment – leads to universal health coverage, health security, improved health status, responsive health systems and equity. The PHC conceptual framework links together all the key concepts related to PHC performance in a logical sequence where health system determinants are needed to support service delivery, which then leads to the expected outcomes and impact. The framework can also be used to develop an analytical framework for measuring the different components.^22^

### Theoretical framework

**FIGURE 2:**
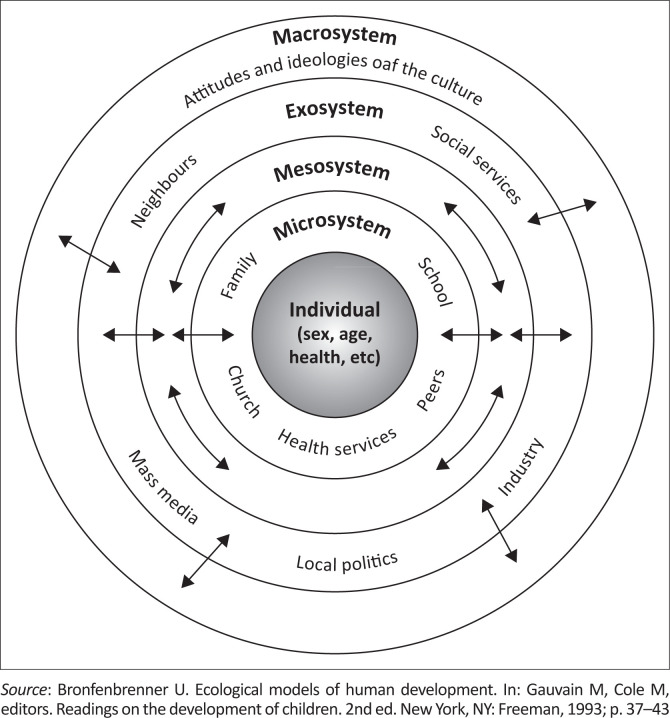
Example of a theoretical framework.

When used, a theoretical framework draws on established theories to guide the formulation of research questions and methods, to provide a deeper interpretation of findings, and to situate findings within broader disciplinary debates. Established theories – such as grounded theory, social learning theory, or systems theory – can provide a deeper explanation of the phenomenon. It is not simply a citation of theory, but an active use of theory to frame the research problem, shape hypotheses or propositions and structure academic argumentation. The theory provides intellectual scaffolding for the methods, analysis and discussion. A thesis can demonstrate how the theoretical framework has guided the design, analysis and interpretation of findings to demonstrate ‘doctorateness’.^21^ Figure 2 illustrates Bronfenbrenner’s ecological systems theory.^23^ This theory was used as a framework in a doctoral thesis that identified risk factors for the use of nyaope, an illicit street drug in Tshwane.

Ultimately, doctoral candidates should demonstrate how they have applied these frameworks and how their integration supports originality and scholarly contribution. Avoiding superficiality and ensuring that each framework is appropriately embedded and justified within the thesis are key indicators of research maturity and scholarly competence.

## Research paradigms and study design

### Research paradigms

While introducing your study design, it is customary to position your work within a particular research paradigm. While doctoral candidates should be aware of philosophical foundations such as ontology (the nature of reality), epistemology (how knowledge can be acquired) and methodology (the approaches used to generate knowledge), in the health sciences, it is uncommon to explore these in great depth. Most Family Medicine theses adopt a pragmatist stance, often employing mixed methods that draw on multiple paradigms. Methodological positioning is usually described in relation to the choice of study design rather than as part of the conceptual or theoretical framework. To make this clearer, we include a separate subsection on research paradigms and study design, which explains how candidates can briefly articulate their philosophical stance, justify their methodological choices and link these to their analytical strategies.^24^

For example, a researcher operating within a positivist paradigm, which assumes a single, observable reality (realist ontology) and objective knowledge (objectivist epistemology), may adopt a theoretical framework such as the Health Belief Model, utilise quantitative methods such as structured surveys, and design a conceptual framework that identifies measurable variables and hypothesised relationships.^25^ In contrast, a constructivist paradigm, which assumes multiple, socially constructed realities (relativist ontology) and knowledge that is interpreted from the participants’ experiences or perceptions (subjectivist epistemology), may draw on interpretive theories such as symbolic interactionism, employ qualitative methods such as in-depth interviews, and develop a conceptual framework that highlights themes, meanings and contextual nuances.^26^

Equally critical is an understanding of research paradigms and their implications for structuring academic arguments. Paradigms offer a lens through which reality is interpreted and provide the foundation for the logic of inquiry. Making one’s paradigm explicit helps to frame the research process and justify choices throughout the study. As Trafford and Leshem emphasise, researchers should be able to explain how their chosen paradigm influenced their formulation of research questions, selection of methods and interpretation of findings. Common paradigms include positivism, interpretivism and critical realism, each with distinct assumptions for research design and analysis. Pragmatism allows flexibility, enabling the use of both qualitative and quantitative methods (mixed methods) where appropriate, with the aim of generating practical solutions to real-world problems. This orientation is well-suited to the applied and multidisciplinary nature of doctoral research in Family Medicine.^21^

### Study design

**FIGURE 3:**
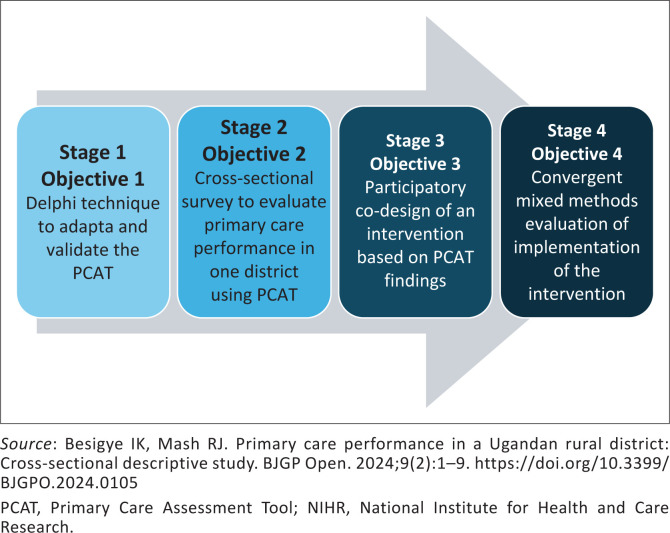
Example of a diagram used to illustrate the study design and alignment with objectives and methods.

A doctoral thesis often contains several study components to address the objectives of the study. These components may require different methodological choices. The study design should explain the different methods to be used, how they relate to the objectives and how they are related to each other. Often, a diagram can assist the reader to understand the study design better, as shown in Figure 3.^27^ This was a doctoral study using the Primary Care Assessment Tool in Uganda.^28^

For mixed methods research, candidates should describe how qualitative and quantitative findings are integrated, as this demonstrates how the strategy enhances understanding of the research problem. The point of integration will depend on the chosen design: in a convergent design, integration typically occurs at the results stage, where findings from both strands are presented together and integrated if possible. In sequential designs (e.g. explanatory or exploratory), integration is often highlighted later in the synthesis chapter, where one strand is used to build upon or explain the other. Regardless of the design, integration should reflect a coherent relationship between data strands.^27^ This is dealt with in another article of this series.

## Analytical frameworks

The study design describes how the research objectives will be addressed through chosen methods, while the analytical framework refers more narrowly to the logic of how data will be examined and interpreted (e.g. thematic analysis, regression modelling). To avoid duplication, many theses integrate this discussion into the methodology chapter, often using a diagram to show how objectives, methods and analytic strategies are connected.^20,24^

The analytical framework, often less discussed, provides the practical logic of how data will be examined. It includes an explanation of what is being analysed (e.g. key variables or constructs), analytic strategies (e.g. thematic analysis, discourse analysis, regression models) and the rationale or justification for this approach. This framework ensures the analysis remains rigorous and aligned with the theoretical lens and research questions.^24^

All these different frameworks may feel overwhelming. Remember that examiners are primarily looking for clarity, coherence and scholarly maturity, rather than the mandatory presence of multiple frameworks.^21,29^

## Developing your synthesis or conclusions chapter

The synthesis chapter is the intellectual pinnacle of the PhD thesis. It is where the authors go beyond reporting their results to critically interpret and integrate their findings in relation to their research questions, theoretical framework and existing literature. It answers the fundamental question: ‘What is the PhD in this PhD?’ This pivotal chapter weaves together the different strands of the research to form a coherent and meaningful contribution to knowledge. It should also address the limitations of your study, propose directions for future research, and, where appropriate, provide practical or policy recommendations. It is also useful to describe the plan to disseminate your work and ensure that it has an impact.

### Structuring the synthesis

In a traditional monograph, the synthesis chapter performs the role of a discussion in a journal article: it summarises the key findings, interprets them in relation to the literature and conceptual or theoretical framework, discusses strengths and limitations and prepares the ground for recommendations and final conclusions. In a thesis by publication, however, the detailed discussion of findings is already contained within the individual articles. The final chapter, therefore, moves directly to integration of the findings at the level of conclusions, recommendations and impact. Additional discussion should only be included if it adds value beyond what is presented in the articles, and repetition should be avoided.

Begin the traditional synthesis chapter by clearly presenting the key findings of your study. The purpose of summarising findings is not to repeat detailed statistical outputs or extensive qualitative themes, as these are already presented in the results chapter. Instead, candidates should distil the main findings into a concise narrative that highlights the key outcomes of the study. In qualitative or mixed methods research, synthesis may be approached thematically or conceptually. A thematic synthesis identifies recurring motifs across datasets to provide a descriptive account. A conceptual synthesis, in contrast, integrates findings at a higher level of abstraction to generate new concepts or theoretical insights.^30^ The choice depends on your study’s epistemological orientation and the intended level of abstraction. Evans, Gruba and Zobel^30^ propose a helpful strategy for structuring the key findings: the ‘mud map’ technique:

- List all key findings from the included studies.
- Group related findings into thematic or conceptual clusters.
- Explore the relationships between clusters.

In a thesis by publication, the final chapter should not reiterate what is already in the articles but start with a synthesis of the findings as a series of conclusions related to each of the objectives.

Whether a traditional thesis or by publication, the synthesis must clearly communicate the study’s contribution to knowledge by making visible the new understanding the research has generated and showing how it adds to or challenges existing knowledge.

A robust traditional synthesis chapter should then move beyond description to engage deeply with the findings through analytical questioning.^31^ This next section of the chapter should interrogate why those findings matter and how they contribute to broader scholarly conversations. This involves examining patterns and exceptions, theorising mechanisms and exploring how the findings relate to your research questions and framework (Table 4).^31^ Using such an approach encourages clarity, depth and coherence across the synthesis chapter. It also helps ensure that the candidate demonstrates intellectual ownership over the research narrative – an expectation in doctoral-level work. In a thesis by publication, this discussion is presented in each of the articles.

**TABLE 4:** Reflective questions to support critical engagement in the synthesis.

Analytical focus	Guiding questions
Relationships and patterns	What relationships exist among the findings? Are there observable trends or anomalies?
Underlying mechanisms	What theoretical or contextual explanations account for these findings?
Engagement with literature	How do the findings support, challenge or extend prior research or theoretical claims?
Alignment with research design	This does not require a separate subsection but should be demonstrated throughout the thesis. Alignment functions as an analytical checkpoint, ensuring that findings relate back to the aims, research questions and chosen framework. In this sense, it parallels the role of the analytical framework, which makes explicit the coherence between methodological choices, findings and their interpretation.
Critical reflexivity	What alternative explanations exist? What are the limitations or tensions in your interpretation?
Contribution to knowledge	What new insights emerge, and how do they advance understanding in your field?

*Source*: Lempriere M. The difference between empirical and discussion chapters (and how to write them) [homepage on the Internet]. The PhD People; 2020. Available from: https://www.thephdpeople.com/structuring-your-phd/the-difference-between-an-empirical-and-discussion-chapter/

### Engaging with theoretical or conceptual frameworks

Whether a traditional thesis or by publication, a strong synthesis chapter considers the conclusions or key findings in relation to the original conceptual or theoretical framework. The framework can provide a valuable way of summarising the findings, while also serving as a point of comparison to identify where adaptations or refinements are needed. In some cases, the findings may even lead to the development of a new framework. In this way, conceptual or theoretical integration is not an abstract requirement but a practical means of demonstrating coherence, scholarly maturity and the contribution of the study to disciplinary understanding.^32^

### Acknowledging strengths and limitations

Transparency about the strengths and limitations of the study is a hallmark of academic rigour. These considerations may be included as a stand-alone section in a traditional thesis or integrated into the discussion section of articles, where they help the reader to interpret the findings. Discuss issues such as sample constraints, methodological challenges or threats to validity. Generalisability or transferability should be discussed. Rather than undermining the study, thoughtful discussion of limitations strengthens its credibility.

### Implications or recommendations

The implications and recommendations that flow from the findings are typically presented in a separate section. Recommendations feel more certain and directive and should be supported by strong evidence; implications feel more tentative and less prescriptive. Here, candidates should consider how their work is relevant to different audiences or stakeholders – including academics, practitioners, policymakers and communities – and present recommendations or implications in ways that are meaningful for each.^29^ Researchers are an important group and candidates should identify unanswered questions and recommend directions for future research that could build on the current work.^33^ Recommendations or implications should clearly flow from and be supported by the findings. Researchers are often tempted to advocate for changes that are not supported by their findings, and these should be avoided, even if they are good ideas or evidence-based.

### Discussing the future dissemination and impact of your work

A final section of the chapter should discuss the knowledge translation plan and how the work will be disseminated and have an impact. A thesis by publication will summarise what has been published and what will be published. Publishing original research articles in peer-reviewed scientific journals and presenting the work at conferences is the traditional approach, but it only engages other academics and researchers. A broader approach to knowledge translation with different stakeholders is discussed in another article in this series.

### Common pitfalls to avoid

Typical pitfalls in synthesis chapters include:

- Over-reliance on a descriptive summary without analysis.
- Disorganised structure or weak thematic coherence.
- Failure to engage with the relevant literature. Candidates should connect their findings to the broader body of knowledge – including theoretical, empirical and methodological work as appropriate – rather than limiting the discussion to a description alone.
- A lack of a clear argument, where findings are not synthesised into a coherent line of reasoning.
- A lack of a clear contribution, where the thesis fails to demonstrate how it advances knowledge, theory or practice in the field.

To avoid these, plan the chapter carefully, maintain critical distance, and ensure every section aligns with your core research questions. Time management is also vital – leave ample time to write, revise and reflect.

## Conclusion

Doctoral writing is an iterative, intellectually demanding process that goes far beyond the mechanical recording of research. It is central to the development of a doctoral candidate’s scholarly identity and is instrumental in establishing their contribution to knowledge. This article has provided practical guidance to support PhD candidates in developing authoritative academic writing and structuring a coherent thesis. From crafting well-reasoned arguments to aligning research frameworks and navigating thesis formats, each element contributes to the overall rigour and clarity of the final product. Special attention was given to the synthesis chapter as the thesis’s intellectual pinnacle, where findings are integrated, critically interpreted and positioned within existing theory and literature. By demystifying the process and clarifying expectations, this guide aims to empower doctoral candidates to write with confidence, clarity and scholarly purpose – ultimately producing research that meets the highest academic standards and has real-world impact.

## References

[1] Kamler B, Thomson P. Helping doctoral students write: Pedagogies for supervision. New York, NY: Routledge; 2014.

[2] Van Schalkwyk S, Jacobs C. 4. Borders and tensions in the context of doctoral writing. In: Badenhorst C, Amell B, Burford J, editors. Re-imagining doctoral writing. Louisville: University Press of Colorado, 2021; p. 89–106.

[3] Mantai L. Feeling like a researcher: Experiences of early doctoral students in Australia. Stud High Educ. 2017;42(4):636–650.

[4] Cotterall S. The rich get richer: International doctoral candidates and scholarly identity. Innov Educ Teach Int. 2015;52(4):360–370. 10.1080/14703297.2013.839124

[5] Xu L, Grant B. Doctoral publishing and academic identity work: Two cases. High Educ Res Dev. 2020;39(7):1502–1515. 10.1080/07294360.2020.1728522

[6] De Magalhães MB, Cotterall S, Mideros D. Identity, voice and agency in two EAL doctoral writing contexts. J Second Lang Writ. 2019;43:4–14. 10.1016/j.jslw.2018.05.001

[7] Ochoa-Pachas JM, Cáceres-López R, Chirre-Castillo EA, Marchinares H, Enrique A, Suárez-Aguilar ZB. Alignment criterion to evaluate research. Philip Roth Stud. 2024;20:2.

[8] Murray R. Ebook: How to write a thesis. Maidenhead, Berkshire: McGraw-Hill Education; 2017.

[9] Winner WE. Descriptive and analytical writing. A handbook for analytical writing: Keys to strategic thinking. Cham: Springer International Publishing, 2013; p. 9–15.

[10] Duschl R. Science education in three-part harmony: Balancing conceptual, epistemic, and social learning goals. Rev Res Educ. 2008;32(1):268–291. 10.3102/0091732X07309371

[11] Barasa D. Demystifying the discourse: Techniques to effective academic writing. J Re Acad Writ. 2024;1(1):13–21. 10.58721/jraw.v1i1.571

[12] Baber WW. Crafting arguments in academic writing. Academe. 2018;1(1):30–40.

[13] Thompson P. Achieving a voice of authority in PhD theses. In: Hyland K, Guinda CS, editors. Stance and voice in written academic genres. London: Palgrave Macmillan UK, 2012; p. 119–133.

[14] University of Pretoria. Faculty of health sciences guidelines for presentation of masters and PhD in a publication format [homepage on the Internet]. 2022 [cited 2025 Jul 09]. Available from: https://www.up.ac.za/media/shared/87/0_0_2025_Updates/ResEthics_New/2025_Guidelines_MAs_and_PhDs_degrees_by_publication_final_apr2025.zp263612.pdf

[15] University of Cape Town. General rules and policies [homepage on the Internet] [PhD degree]. Cape Town; 2024 [cited 2025 Jul 09]. Available from: https://uct.ac.za/sites/default/files/media/documents/PhD_Degree_General_Rules_2024.pdf

[16] Stellenbosch University. Some guidelines for thesis and dissertation layout [homepage on the Internet]. Cape Town; 2019 [cited 2025 Jul 09]. Available from: https://www.sun.ac.za/english/research-innovation/Research-Development/Documents/Generic%20guidelines%20for%20thesis%20and%20dissertation%20layout%20PG%20Skills%20Updated%20Jan%202019.pdf

[17] University of KwaZulu-Natal. Approved college of health sciences guidelines for presentation of thesis [homepage on the Internet]. Durban; 2015 [cited 2025 Jul 09]. Available from: https://doeh.ukzn.ac.za/Libraries/OHdocs/Approved_CHS_Guidenlines_for_presentation_of_thesis_20_August_2015.pdf

[18] University of the Witwatersrand. Faculty of health sciences style guide for theses, dissertations and research reports [homepage on the Internet]. Johannesburg; 2016 [cited 2025 Jul 09]. Available from: https://www.wits.ac.za/media/wits-university/faculties-and-schools/health-sciences/research-entities/documents/FACULTY%20OF%20HEALTH%20SCIENCES%20STYLE%20GUIDE%20FOR%20THESES-DISSERTATIONS-AND-RESEARCH%20REPORTS-Updated%20Marh%202016.pdf

[19] Frick L. PhD by publication – Panacea or paralysis? Afr Educ Rev. 2019;16(5): 47–59. 10.1080/18146627.2017.1340802

[20] Luft JA, Jeong S, Idsardi R, Gardner G. Literature reviews, theoretical frameworks, and conceptual frameworks: An introduction for new biology education researchers. CBE Life Sci Educ. 2022;21(3):rm33. 10.1187/cbe.21-05-0134 35759629 PMC9582830

[21] Trafford V, Leshem S. Doctorateness as a threshold concept. Innov Educ Teach Int. 2009;46(3):305–316. 10.1080/14703290903069027

[22] World Health Organization. Primary health care measurement framework and indicators: Monitoring health systems through a primary health care lens. Geneva: World Health Organization; 2021.

[23] Bronfenbrenner U. Ecological models of human development. In: Gauvain M, Cole M, editors. Readings on the development of children. 2nd ed. New York, NY: Freeman, 1993; p. 37–43.

[24] Pacheco-Vega R. Writing theoretical, analytical and conceptual frameworks [homepage on the Internet]. 2018 [cited 2025 Jul 05]. Available from: https://www.raulpacheco.org/2018/09/writing-theoretical-frameworks-analytical-frameworks-and-conceptual-frameworks/

[25] Park YS, Konge L, Artino Jr, AR. The positivism paradigm of research. Acad Med. 2020;95(5):690–694. 10.1097/ACM.0000000000003093 31789841

[26] Nugroho KY. Constructivist learning paradigm as the basis on learning model development. J Educ Learn. 2017;11(4):410–415. 10.11591/edulearn.v11i4.6852

[27] Besigye IK, Mash RJ. Primary care performance in a Ugandan rural district: Cross-sectional descriptive study. BJGP Open. 2024;9(2):1–9. 10.3399/BJGPO.2024.0105 PMC12421270 39505399

[28] Creswell JW, Clark VLP. Designing and conducting mixed methods research. Los Angeles: Sage; 2017.

[29] Trafford V, Leshem S. Stepping stones to achieving your doctorate: By focusing on your viva from the start. Glasgow: McGraw-Hill Education; 2008.

[30] Barnett-Page E, Thomas J. Methods for the synthesis of qualitative research: A critical review. BMC Med Res Methodol. 2009;9(1):59. 10.1186/1471-2288-9-59 19671152 PMC3224695

[31] Lempriere M. The difference between empirical and discussion chapters (and how to write them) [homepage on the Internet]. The PhD People; 2020 [cited 2025 Jul 12]. Available from: https://www.thephdpeople.com/structuring-your-phd/the-difference-between-an-empirical-and-discussion-chapter/

[32] Booth W, Colomb G, Williams J, Bizup J, Fitzgerald W. The craft of research. The London: University of Chicago Press; 2016.

[33] Silverman D. Doing qualitative research. London: SAGE Publications Ltd; 2021.

